# Effectiveness of a Randomized School-Based Intervention Involving Families and Teachers to Prevent Excessive Weight Gain among Adolescents in Brazil

**DOI:** 10.1371/journal.pone.0057498

**Published:** 2013-02-25

**Authors:** Diana B. Cunha, Bárbara da S N de Souza, Rosangela A. Pereira, Rosely Sichieri

**Affiliations:** 1 Department of Social Medicine, State University of Rio de Janeiro, Rio de Janeiro, Brazil; 2 Department of Nutrition, Federal University of Rio de Janeiro, Rio de Janeiro, Brazil; Universidad Peruana de Ciencias Aplicadas (UPC), Peru

## Abstract

**Objective:**

To evaluate the effectiveness of a school-based intervention involving the families and teachers that aimed to promote healthy eating habits in adolescents; the ultimate aim of the intervention was to reduce the increase in body mass index (BMI) of the students.

**Design:**

Paired cluster randomized school-based trial conducted with a sample of fifth graders.

**Setting:**

Twenty classes were randomly assigned into either an intervention group or a control group.

**Participants:**

From a total of 574 eligible students, 559 students participated in the study (intervention: 10 classes with 277 participants; control: 10 classes with 282 participants). The mean age of students was 11 years.

**Intervention:**

Students attended 9 nutritional education sessions during the 2010 academic year. Parents/guardians and teachers received information on the same subjects.

**Main Outcome Measurement:**

Changes in BMI and percentage of body fat.

**Results:**

Intention-to-treat analysis showed that changes in BMI were not significantly different between the 2 groups (β = 0.003; p = 0.75). There was a major reduction in the consumption of sugar-sweetened beverages and cookies in the intervention group; students in this group also consumed more fruits.

**Conclusion:**

Encouraging the adoption of healthy eating habits promoted important changes in the adolescent diet, but this did not lead to a reduction in BMI gain. Strategies based exclusively on the quality of diet may not reduce weight gain among adolescents.

**Trial Registration:**

Clinicaltrials.gov NCT01046474.

## Introduction

The prevalence of overweight and obesity among Brazilian adolescents has tripled since 1970, and this reached 20.5% in 2009 [1]. Obesity is a chronic process which is associated with a range of diseases; thus, special attention should be paid to its prevention. Besides being related to metabolic disorders, excessive weight gain during adolescence is also a risk factor for extreme obesity in adulthood [2]. According to Schmidt et al. [3], recent socioeconomic changes in Brazil have seen a shift towards obesity among the poor, which is probably related to an increased intake of unhealthy foods.

A National Survey of Schools in Brazil (PeNSE) on food consumption and behavior among adolescents revealed high levels of consumption of unhealthy dietary items such as sugar-sweetened beverages, sweets, and sweet cookies; this was accompanied by a lower consumption of fruits and vegetables. Thus, there is a need for health promotion activities aimed at young people [4].

Schools are considered important places for promoting intervention in order to prevent excessive weight gain, mainly among children; a meta analysis revealed that school-based interventions can improve dietary and physical activity behavior in low- and middle-income countries and that 8 of 12 interventions had a statistically significant effect on BMI [5]. Conversely, a meta-analysis conducted by Khambalia et al., in 2012 [6] combined the findings of 3 meta-analysis and 5 systematic reviews and concluded that the effectiveness of such interventions was yet to be determined. Previous studies are heterogeneous, but significant weight reductions in children have been observed for long-term interventions that combine diet, physical activity, and a family component [7], [8]. For the school interventions targeting changes in food intake, behavior change is frequently observed. However, few studies have demonstrated the impact of these dietary changes on changes in BMI [9]. Nevertheless, a meta-analysis of obesity interventions among children from minorities in the U.S. observed that interventions with a greater number of components had a higher mean effect size [10]. The participation of parents and teachers also appears to be important in terms of changing adolescent behavior [11].

Therefore, the purpose of the present school-based intervention involving the participation of parents and teachers was to promote healthy eating habits among adolescents. The intervention’s ultimate goal was to bring about changes in the BMI of students.

## Subjects and Methods

### 1. Study Design and Setting

We conducted a paired randomized school-based trial in a sample of fifth graders from public schools in the municipality of Duque de Caxias, Rio de Janeiro, Brazil. The study was conducted and reported according to the CONSORT guideline for cluster-randomized trials [12]. The protocol for this trial and supporting CONSORT checklist are available as supporting information; see Checklist S1 and Protocol S1.

The trial was labeled PAPPAS, which stands for “parents, students, and teachers for healthy eating” in Portuguese. The invention focused on positive messages related to the intake of water, fruits, rice, and beans. It also included messages regarding eating fewer cookies, sugar-sweetened beverages, and savory snacks. These items were chosen because of their importance in the dietary habits of adolescents in Brazil [4], [13].

The municipality of Duque de Caxias (population, 842,686 [14]) is in the metropolitan area of Rio de Janeiro, 27 km from the state capital. It is also one of the poorest areas in Brazil. For this study, we chose one of the Duque de Caxias districts, Campos Elíseos (population, 220,000). This district has 35 municipal schools, and we selected 20 schools with fifth grade classes; these were all located in areas not considered high risk for violence. Our sample included most of public schools from Duque de Caxias, and the dropout rate was low.

### 2. Sample Size and Randomization Procedure

A sample size of 444 children (222 in each study arm) was calculated based on 80% power and a significance level of 5%, using a standard deviation of 3 units [15]. We anticipated a difference in BMI of 0.8 kg/m^2^, which corresponded to the increase in the BMI value at the 85^th^ percentile from the ages 10–11 years [16]. As the classes contained 25–30 children, the sample included 20 classes from 20 schools (1 class in each school). There were 10 classes in the intervention group and 10 in the control group. The paired randomization procedure was carried out with schools based on the prevalence of excessive weight (overweight+obesity) in the year preceding the survey; those schools with the closest excess weight scores were matched and then randomly allocated. Each pair in the ranking sequence was randomly drafted with 1 class being assigned to the experimental group and 1 to the control group, using opaque envelopes, in the presence of three investigators. Although cluster sampling requires a greater sample size, the repetition of 3 anthropometric measurements increased the power of the study [17].

### 3. Intervention Classes

The intervention focused on encouraging students to change their eating habits and food consumption over 9 months (from March to November) in the school year of 2010. Trained nutritionists gave monthly 1-h sessions in the classrooms. These sessions included playing games, staging of theater sketches, watching movies and puppet shows, and writing and drawing contests.

The activities were designed to discourage students from consuming sugar-sweetened beverages and sugar as well as getting them to replace snacks based on processed food (especially cookies) with fresh fruits or healthy homemade food. To reinforce the message of the activities carried out with children during the class intervention’s nutritional sessions, a set of messages were sent to the families in the form of illustrated booklets and recipes. The families also received small gifts such as buttons and magnets. In addition, teachers were encouraged to work with the children on the topics addressed in each intervention session. Thus, a set of exercises and suggestions for class work were provided to them. Each session included: (1) activities, related to the subject, to be conducted at the school; (2) folders explaining the intervention program and suggesting the participation of the family, such as reducing purchase of sodas and increasing the purchase of fruits, to be sent home; and (3) strategies for reinforcement of themes by the teachers, using exercises prepared for this purpose, such as specific popular histories or math games.

Briefly, the themes of the intervention sessions were as follows: (1) healthy eating, (2) native Brazilian eating habits, (3) excessive sugar in processed food, (4) marriage of the rice and beans, (5) the beauty of fruits, (6) super water: a super-hero, (7) cookies, (8) mini-market, and (9) food advertisements. A complete description of each session is available at http://www.nebin.org/artigos/Interventions%20-%20PAPPAS.pdf.

The intervention was based on the action-oriented “Pedagogy of the Oppressed” approach developed by the pedagogue Paulo Freire. This approach proposes a new relationship between teachers, students, and society in which individuals learn to cultivate their own growth through situations from daily life that provide useful learning experiences [18]. All 9 intervention sessions explained the concepts on healthy and unhealthy eating habits to students. Students were also given information on barriers to eating healthy items, and they were asked to re-interpret situations based on their own experiences. This involved writing advertisements, participating in a puppet theater, writing about different fruits, etc. For example, in the session on the beauty of fruits, a focal group identified the pros and cons of specific fruits, looked at how children generally consumed them, and examined why some fruits such as bananas were not consumed frequently despite their high availability at school.

The control group received a one-hour section of orientation on general health and advice on healthy eating, at the end of the study.

### 4. Measurements

Although intervention classes were developed over 9 months, data collection consisted of three waves of measurement (baseline, mid-year school (month 6), and post-intervention (end of term). In each of the three waves, anthropometric examination (Body Mass Index and percentage of total body fat) and consumption of food and beverages using a food frequency questionnaire were collected. At baseline and at the end of the study two 24-h recalls were also obtained.

The primary outcome measure was the adolescent body mass index (BMI calculated as weight (kg) divided by square of the height (m). The secondary outcome was the percentage body fat.

Weight was measured using a portable electronic scale (Tanita BC-558, Japan) with a capacity of 150 kg and a precision of 100 g. Height was measured using a portable stadiometer (Alturaexata, Brazil) with an amplitude of 200 cm and variation of 0.1 cm. A maximum variation of 0.5 cm between the 2 measures was admitted; if the difference exceeded this value, measurements were repeated. The mean value of 2 valid measures of height was used. Subjects were weighed and measured with minimal clothing and no shoes following the recommendations of Gordon et al. (1988) [19].

The percentage of total body fat (%BF) was estimated by bioimpedance analysis. This measurement was carried out with a multifrequency tetrapolar bioimpedance device (Tanita BC-558), the same instrument used to measure weight. For the measurements, adolescents were barefoot and upright on the machine while holding a handgrip in each hand [19]. The %BF values displayed by the scale were used. These were defined according to sex and age.

Anthropometric measures were analyzed by Z-scores of BMI-for-age and sex from the distributions of the World Health Organization (WHO) [20] using the Anthro-Plus software [21]. Nutritional status was classified according to WHO-recommended cutoffs [22].

#### Compliance with the intervention

The consumption of food and beverages was assessed at the three moments of assessment, with a food frequency questionnaire (FFQ). The items evaluated were the same from a previously validated FFQ for adolescents in the metropolitan region of Rio de Janeiro, Brazil [23], and included the following intervention items: cookies, sugar-sweetened beverages (including sodas and juices), and beans; it also included several items commonly consumed by adolescents such as fast food (consumed in restaurants or at street stalls) and milk. Further, nutritionists conducted face-to-face interviews with the students at schools to obtain two 24-h recalls (24HR), one at baseline and one at the end of the study; the Multiple Pass Method was used [24].

To verify compliance with the proposed intervention, we asked parents/guardians about their stages of motivation in terms of changing food behavior twice, i.e., at baseline and at the end of the study. The 5 options were as follows: (1) I am not thinking about changing my diet; (2) I am thinking about changing my diet, but I am not sure; (3) I have decided to change my diet, and I am thinking about how to do this; (4) I am changing my diet, but I am having difficulties; and (5) I am changing my diet successfully. These responses parallel the 5 stages of change (precontemplation, contemplation, decision, action, and maintenance) in the Transtheoretical Model [25].

The present study was conducted according to the Declaration of Helsinki guidelines, and all procedures involving human subjects/patients were approved by the Ethics Committee of the Institute of Social Medicine (State University of Rio de Janeiro, Brazil). Written informed consent was obtained from all the participants’ parents. Additionally, a short questionnaire containing information on food consumption and the intention to change eating habits was sent to parents/guardians at baseline (with the terms of consent) and at the end of the study.

### 5. Data Analysis

Because of the paired cluster design used when allocating schools, we used mixed models to perform a random effects meta-analysis technique that included all data [26]. This analysis took into account both the within-school and between-school components of variance. To carry out this procedure, a school-pair estimator was created and was used in the PROC MIXED procedure of SAS v 9.3 (Statistical Analysis System).

These mixed models also allowed for the exploration of factors associated with body weight variation in multilevel analysis where the class was on the second level and the measures taken were on the first level. The models used considered both missing data and the cluster effect [27].

For the food and drink intake, data were categorical/dichotomous, and the PROC GLIMMIX procedure in SAS was performed.

## Results

Of the 574 students enrolled in this study (281 in the control group and 293 in the intervention group), 478 (83%) returned consent forms signed by parents/guardians at the first follow-up of the study and had their measurements taken. All students in the intervention group, who were present in the classroom, participated in the nutritional education sessions.

**Figure 1:**
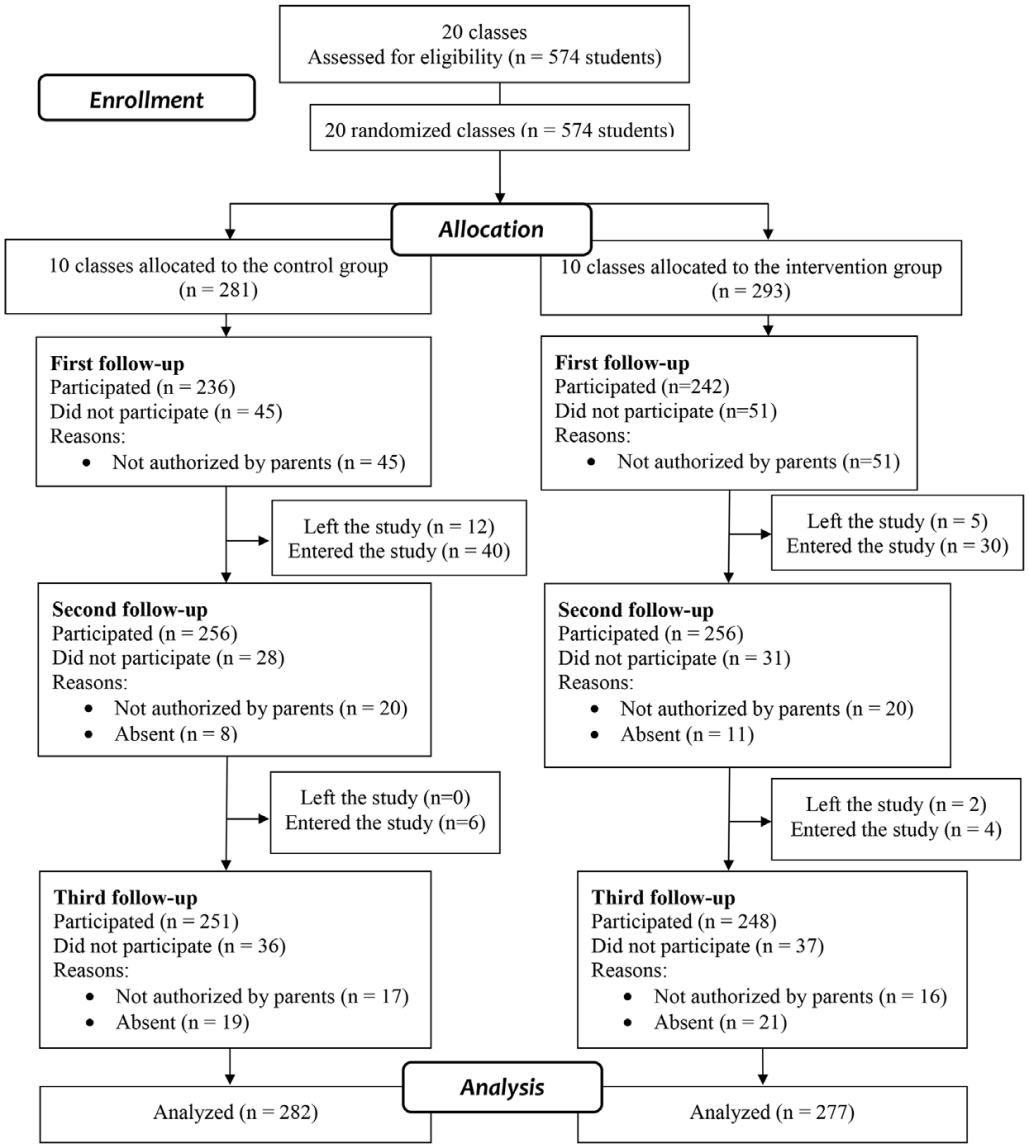
Progress of individuals during the study.

During the school year, a number of students left the school and others joined. In addition, some students who did return the signed informed consent at baseline did so in the middle of the school year (phase 2) or during the third phase of the study. Figure 1 shows the flow of participants throughout the study (Figure 1).

There were no differences between the control and intervention groups at baseline for age, race, and sex. However, for anthropometric measures, there was a higher prevalence of overweight and obesity among participants in the control group. Accordingly, weight, height, and BMI were higher in the control group. At baseline, the intake of beans, cookies, and sugar-sweetened beverages as evaluated by the 24HR was slightly higher in the control group. Conversely, participants in the intervention group consumed more fruits (Table 1).

**Table 1:** Anthropometric characteristics and intake of selected food items at baseline according to group allocation (N = 478).

	Control group (n = 236)		Intervention group (n = 242)	
	Mean	SD	Mean	SD
Age (years)	11.2	1.3	11.2	1.3
Weight (kg)	39.5	10.4	36.9	9.9
Stature (cm)	145.0	8.4	144.5	9.4
BMI (kg/m2)	18.6	3.7	17.4	3.0
Fat %	22.7	6.8	21.4	5.9
Bean consumption (g/day)*	256	212	244	199
Fruit consumption (g/day)**	52	105	76	169
Cookies consumption (g/day)	73	108	58	77
Sugar-sweetened beverage consumption (ml/day)	446	411	430	350
Skin color (%)				
White	25.7		25.6	
Brown	42.7		47.6	
Black	31.5		26.8	
Male (%)	51.4		52.3	
Nutritional status				
Underweight (%)*	2.1		4.1	
Overweight (%)*	17.4		13.6	
Obesity (%)*	13.2		5.4	

* p-value≤0.05.

** p-value <0.01.

Due to baseline unbalances for BMI, fruit, and bean consumption, analyses with and without adjustment for baseline measures were conducted (Table 2). A statistically significant change in BMI over time was observed (variable time *intervention; p = 0.02), indicating increase in BMI in the intervention compared to control group, when model includes baseline variables. Modeling without adjustment for baseline showed no association. Main results are presented using the results from models without baseline adjustment since adjustment in randomized studies have been criticized as presented in the discussion section. Therefore, we concluded that there was no differences in the BMI changes between the intervention and control groups (β = 0.003; p-value = 0.75) (Table 2). Further, the %BF did not change significantly in the 2 groups as a result of the intervention (p = 0.93; mixed model effects). Furthermore, the prevalence of overweight and obesity did not change as a result of the intervention; overweight for both the intervention and control groups was 17% at baseline and 14% after 1-year follow-up. Obesity increased from 13% to 16% in the control group and from 5% to 8% in the intervention group.

**Table 2:** Variation in mean BMI over time comparing intervention with control group (N = 559).

	Model without adjustment for baseline measures		Model adjusted for baseline measures*	
	β	p-value	β**	p-value
Age	0.033	<0.01	0.004	<0.01
Intervention/control group	−0.069	0.02	−0.006	0.30
Time	0.013	<0.01	0.015	<0.01
Time* Intervention/control group	0.003	0.75	0.006	0.02

* Adjusted for BMI, fruit and bean consumption at baseline.

** Coefficient associated with group*time based on proc mixed procedure in SAS.

Measures of compliance using the frequency of food consumption indicated a statistically significant reduction in the frequency of daily consumption of cookies and sodas and an increased frequency of consumption of fruits in the intervention group, compared with that of the control group (Table 3). Similar results were observed for the 24HR data (data not shown).

**Table 3:** Variation in daily frequency of cookies, sodas, juices, beans and fruits after the nine months intervention (N = 559).

	Control Group		Intervention Group		β*	p-value
	Mean	95% CI	Mean	95% CI		
Cookies	0.02	−0.04–0.09	−1.35	−0.20-−0.75	0.13	<0.001
Sodas	−0.08	−0.18–0.02	−0.20	−0.30-−0.11	0.13	0.02
Juices	0.01	−0.12–0.14	−0.16	−0.28-−0.04	0.03	0.66
Beans	0.00	−0.35–0.04	0.01	−0.02–0.05	−0.01	0.54
Fruits	0.10	−0.05–0.26	0.17	0.01–0.34	−0.16	0.04

* coefficient associated with group*time based on proc glimmix procedure in SAS.

The intra-class correlation coefficient for BMI was 0.07, indicating a small cluster effect.

Parents’ motivation to change eating habits increased over time. At baseline, 37% of parents/guardians reported that they were thinking about changing their diets, while at the end of the study, this percentage had dropped to 33%. Furthermore, the proportion of parents/guardians who reported that they had successfully changed their diets increased from 7% to 11% between baseline and the end of the study.

## Discussion

In this community trial involving municipal school fifth graders in a poor neighborhood in Brazil, no reductions in BMI gain were observed despite involving family, teachers, and school children. This is also despite the fact that the intervention group changed their food habits in accordance with the intervention. Thus, our findings are consistent with the results of other school-based interventions, which focused on changes in BMI of adolescents [28]–[36].

Therefore, we need to ask why proposals aimed at improving eating habits towards healthy items are not successful in reducing excessive weight gain. Our data showed a high baseline intake of sodas and cookies with mean values of approximately 400 mL and 70 g per day, respectively, and although there were statistically significant reductions in the consumption of these items as a result of our intervention, these were relatively small, especially in terms of soda intake. In terms of the proposed increase in the consumption of fruits, the frequency had increased but was still far lower than at least 1 portion of fruit per day. An increase in the consumption of healthy items without a decrease in eating unhealthy items may not lead to reduced energy intake.

In our study, a higher baseline BMI was observed in the control group. Although such findings are often prone to measurement errors, it is still unclear if the baseline measurement error should be adjusted for in this context. A study based on computer simulations clearly indicated that in randomized controlled trials, adjusting for baseline unbalances leads to bias, whereas the ordinary least squares estimator without adjustment for measurement error is unbiased [37]. Therefore, we observe a statistically significant association in the variable change BMI over time when modeling includes baseline imbalanced variables and no association in the modeling without adjustment for baseline measures. These finding are in line with the concern that by forcing a baseline balance, a spurious relationship between treatment and outcome can be observed [38]. As an example, unbalances towards greater prevalence of overweight in the experimental group would have indicated a greater reduction in BMI in experimental compared to control.

Our results are consistent with findings of systematic reviews of fruit intake and obesity, which indicate that increased fruit intake does not lead to a displacement of other items, allowing for a reduction in weight gain [39], [40]. In addition, data from a large national budget survey in Brazil showed that households that had a greater number of healthy items also had high availability of sugar and other unhealthy items [41].

Therefore, our data may corroborate other studies indicating that a reduction in energy intake may be difficult to accomplish by simply promoting healthy eating and that replacement by healthy food items may not completely displace more obesogenic items.

Conversely, the strength of this study is the positive individual changes that were observed in the food intake of the students, family members, and teachers involved in the intervention. This was observed even in the absence of major environmental interventions in the scope of the study.

Among the many programs developed with schools in order to prevent obesity, some have achieved good results in the behavioral changes of children with regard to eating habits [42],[43] but have led to little or no effect on the variation of weight [9], [42], [44], [45].

Lubans et al. (2012) [46] conducted a 12-month multicomponent school-based obesity prevention program in low-income communities in Australia, including teacher professional development, school sport sessions, nutrition workshops, parent newsletters, and text messaging for social support; which also failed to significantly reduce the increase in BMI. However, they observed changes in body composition.

Also, the “New moves”, a preventing weight-related problems group-randomized study in adolescent girls, conducted in Minnesota, did not lead to significant changes in BMI, but improved eating patterns, sedentary activity, unhealthy weight control behaviors, and body/self-image [33].

Our decision to involve family members, teachers, and children was an attempt to increase behavioral changes and make an impact on weight gain. Parents are generally responsible for determining the supply of food within households; in addition, parental influence is associated with the development of sustainable food practices among children, as indicated by effectiveness in trials of prevention/treatment of obesity when the least 1 parent is involved [42], [47]. Further, educators have an important role in supporting healthy choices made by children. These educators should be actively involved in intervention strategies based on nutritional education to ensure greater levels of success [48]. In our study, we observed an increase in parents’ motivation to change dietary habits levels during the school year, which was probably important in reinforcing the adoption of healthy habits by children.

In studies such as the present one, there is possible bias in reporting towards the expected change in behavior in the experimental group; however, we avoided this by using two methods to provide this information (FFQ and 24HR). Both instruments were employed for measuring changes in eating behavior, and we found that there was a positive change in terms of our intervention goals for the consumption of fruits, cookies, and sugar-sweetened beverages. Only the intake of beans showed no change; however, the reported frequency and portion of intake was already high at baseline. In this study, we have presented the results obtained by the FFQ because this instrument captures the usual intake of individuals. It would be expected that the 24HR provided a less biased report compared with the frequency questionnaire, and we found similar values and positive change with both methods. However, both are dependent on memory, and this is a possible limitation.

Another possible limitation of the study is the fact that the participant satisfaction was not measured; however focus groups were conducted before the beginning of activities, with the aim of discovering the preferences of children/adolescents.

Randomization process did not follow completely the consort guideline and was conducted by the investigators, but it was done for each school, using opaque envelops in the presence of three investigators.

Despite the failure of our intervention to bring about an overall change in the students’ BMI, the results of this study are important. This is because the dietary patterns of Brazilian adolescents are characterized by low consumption of vegetables and fruits and high consumption of sweets and sodas as well as foods high in sodium [4], [12], [49], [50]. Importantly, our results indicate that these behaviors can be changed. Data from the Brazilian Dietary Survey, which was conducted by the Brazilian Institute of Geography and Statistics in conjunction with the latest Household Budget Survey, showed that Brazilian adolescents consume 4 times more filled cookies (12.3 g/day) than adults (3.2 g/day). They also drink a greater amount of soft drinks and other sugar-sweetened beverages. The average daily intake of sugars is also higher among teenagers, i.e., approximately 110 g per day [51].

While it is true that a reduction in obesity in the population will not be achieved by 1 or 2 isolated interventions, it is necessary to find effective strategies, especially among economic groups with lower purchasing power. Possible strategies to be explored in communities such the one in this study include an increase in intensity and duration of the interventions, integrating physical activity and/or sedentary behavior, and combining school activities with environmental changes such as reducing the easy access to sodas and low price of junk food.

Primary prevention of obesity is critical in developing countries because of a strong association between overweight and lower social class. Further studies using multicomponent intervention strategies should be able to increase consumption of healthy dietary items, which are associated with a significant reduction of total energy intake from items such as sodas and cookies. Another good strategy would be to incorporate classroom-based physical activities in such interventions. This is particularly important in low income schools, such as the ones in the study, where outside exercise and games areas are not available.

When designing this study, we had anticipated that we could change the food environments at schools by discouraging the sale of unhealthy foods at school canteens. However, before the intervention began, most of the participating schools had already closed their canteens in line with a municipal policy. Nonetheless, many students still brought processed salty snacks, filled cookies, and sodas to school. In addition, physical activities were not possible because of lack of support from principals and teachers. More importantly, there school facilities were inadequate; only 2 of the 20 schools had a sports field.

Lastly, although several studies conducted in developed countries have shown an association between lower consumption of soft drinks and a reduction in weight gain, this was not the case in Brazil. This is because soft drinks are substituted by juices containing sugar in this country [52]. Thus, further studies are required to develop messages and strategies that not only change specific eating behaviors but also lead to reductions in weight gain, especially among low socio-economic groups.

## Supporting Information

Checklist S1 **CONSORT Checklist.**
(DOC)

Protocol S1 **Trial Protocol.**
(DOC)

Doc S1 **Approval of the Ethics Committee.**
(JPG)

Doc S2 **Description of the intervention sessions.**
(DOC)
