# Supplementary material for: Effectiveness of a Randomized School-Based Intervention Involving Families and Teachers to Prevent Excessive Weight Gain among Adolescents in Brazil
Source: PLoS One. 2013 Feb 25;8(2):e57498. doi: 10.1371/journal.pone.0057498 (PMC3581462; doi:10.1371/journal.pone.0057498)
Supplement: Protocol S1 — Trial Protocol. (DOC) [file pone.0057498.s002.doc]

(applicant institution) Social Medicine Institute, State University of Rio de Janeiro

(***date) May 28, 2008_***

**Preventing Excessive Weight Gain by Reducing Carbonated Beverage and Sugar Consumption and Increasing Physical Activity among Public School Adolescents from the Metropolitan Area of Rio de Janeiro**

1. **Project Background and Rationale**

Obesity is a major public health problem in many countries throughout the world, mainly among the young population and the poor (Basset & Perl, 2004). In Brazil, obesity and overweight have been increasing among adults (Monteiro et al., 2004) as well as in adolescents (Veiga et al., 2004). Overweight has tripled since the 1980s, reaching 17% of the Brazilian teenagers in 2003 (IBGE, 2006). The effects of weight gain in addition to inadequate food habits have been observed in studies of Brazilian teenagers, which revealed alterations in serum lipids and blood pressure (Alvarez et al., 2006) Oliveira et al., 2001). Dietary interventions that could prevent excessive weight gain during childhood and adolescence are fundamental to curb this epidemic.

Weight gain and obesity result from the conjunction of factors related to individual biological characteristics, behavior and environment (Thomas, 2006). Individual biology and genetics are not modifiable. Behavioral features include habits, emotions, attitudes, and beliefs, while environmental issues are connected to macro-social determinants such as economics, education, work, health assistance, etc., and also to micro-social factors (such as access to facilities for physical activity, to information, etc.). Intervention aiming to change the progress of overweight and obesity should include both behavioral and environmental factors.

The school years are a strategic period for modifying behaviors such as food choices and physical activity, and school is considered a suitable setting for the promotion of healthy lifestyles (Warren et al., 2003) since most urban children are in school, even in the poorest areas. Also, activities in school avoid stigmatization since they target all children (Thomas, 2006). Although some educators think that schools already have too many responsibilities and that prevention of obesity should not be an additional concern (Newby et al., 2004), school is still considered to be an important element in the effort to prevent childhood obesity (Ludwig et al., 2001). Besides, in countries such as Brazil, public schools are one of the few public institutions that reach the majority in the low socioeconomic groups.

Many programs related to obesity prevention in schools have been an aid mainly to children that already present overweight problems, and scarcely turn into a real prevention program. Furthermore, many prevention studies have found that positive changes in knowledge regarding healthy foods do not translate into significant changes in weight gain in the students (Stice et al., 2006). Therefore, of the many programs tested among school children in order to prevent obesity, few have achieved statistically significant results on prevention of weight gain (Atkinson & Nitzke, 2001). Usually, these initiatives include physical activities and nutritional behaviors, and most of them have attained good results in educating children on these issues (Gortmaker et al., 1999; Stice et al., 2006), but with little or no effect on weight change (Stice et al., 2006; Katz et al., 2007).

Certainly, reduction of obesity in the population will not be achieved with one or two initiatives alone, but it is necessary to find strategies that may show efficacy, mainly among low socioeconomic groups. Also, primary prevention of obesity should be mandatory in developing countries given the burden associated to chronic diseases related to obesity. In addition, unhealthy dietary patterns are not a privilege of overweight individuals. In a population based sample of adolescents in Rio de Janeiro, overweight and normal weight adolescents had similar intake of many types of foods, with both groups eating less than the recommended amounts for fruits, vegetables, dairy, and beans (Andrade et al., 2003). Therefore, youths as a group, regardless of their nutritional status, need to adopt healthy eating habits and increase physical activity.

A randomized trial with British school children with focus on reduction in the consumption of carbonated beverages showed a statistically significant and clinically relevant reduction in weight gain in the experimental group compared to control (James et al., 2004). Rationale of the study was to reduce overall caloric intake by the substitution of sodas for non-caloric drinks, and many strategies were developed to incorporate this message. The result of the British study (James et al., 2004) is particularly important for developing countries, since it was based on sugar-sweetened carbonated beverages alone. The result of this intervention can be more appealing for public health programs compared to the usually more successful school interventions, which are comprehensive in nature (Katz et al., 2007), and harder to be implemented in most countries.

Soda intake has grown more than 400% in the last three decades in Brazil (Levy-Costa et al., 2005) and there are now many low cost brands of soda available for the low income population. A free, healthy lunch is served daily at public schools, but most schools have cafeterias where carbonated and sweetened beverages are sold. In the city of Rio de Janeiro, Brazil, intake of sodas increased from 28 ml per capita/day in 1975, to 103 ml in 1996 (Sichieri, 1998).

A specific physiological role on regulation of energy intake while ingesting liquids indicates that people are more prone to ingesting greater amounts of energy from liquids than from solid foods (Van Wymelbeke et al., 2004). Nevertheless, soda and juice intake was not associated to weight gain in a cohort of school children of low socioeconomic level (Newby et al., 2004). Conversely, other longitudinal studies conducted in the United States have observed increases in BMI (Body Mass Index=weight/stature^2^) and in the prevalence of overweight associated to soda consumption (Ludwig et al., 2001; Berkey et al., 2004; Striegel-Moore, 2006).

A meta-analysis review of sugar-sweetened carbonated beverages linked their intake to weight gain in both children and adults (Malik et al., 2006), but in a large national survey – UK National Dietary and Nutritional Survey of Young People – using weighed 7-day food records, it was observed that the risk associated with caloric soft drinks was non-linear, with an increased risk only for very high consumers ([Gibson &](http://www.ncbi.nlm.nih.gov/sites/entrez?Db=pubmed&Cmd=Search&Term="Gibson S"%5BAuthor%5D&itool=EntrezSystem2.PEntrez.Pubmed.Pubmed_ResultsPanel.Pubmed_RVAbstractPlus) [Neate, 2007)](http://www.ncbi.nlm.nih.gov/sites/entrez?Db=pubmed&Cmd=Search&Term="Neate D"%5BAuthor%5D&itool=EntrezSystem2.PEntrez.Pubmed.Pubmed_ResultsPanel.Pubmed_RVAbstractPlus).

Intake of sodas by American students is influenced by friends and parents’ soda intake (Grimm et al., 2004), and prospective studies have shown a positive effect of changes in family lifestyle on reduction of obesity in children (McGarvey et al., 2004). Educators have also an important role in supporting children’s health choices. For example, positive changes in students’ cardiovascular risk factors were obtained by including health topics in school activities with participation of the teachers (Johnson et al., 2003).

Therefore, the school appears to be an adequate environment to test the role of discouraging the intake of sodas and sweetened beverages as a way to prevent excessive weight gain. However, a recent trial carried out in the municipality of Niteroi, located in the Rio de Janeiro metropolitan area, showed that focusing only on sweetened carbonated beverages may not be effective, since a statistically significant small decrease in the daily consumption of carbonated drinks in the intervention compared to control schools was not followed by a significant overall reduction in BMI. Only students who were overweight at baseline showed greater BMI reduction (-0.4 kg/m^2^ in the intervention group compared to -0.2 kg/m^2^ in the control group (p=0.11)), and this difference was statistically significant among girls (p=0.009). In addition, fruit juice consumption was slightly increased in the intervention group (p=0.08), although not among girls (Sichieri et al. in press). In the United States, (Cavadini et al., 2000) also found that a small reduction in soda consumption was substituted for a greater intake of juices with great addition of fructose. *In this proposal, the exchange between sweetened beverages will be prevented by stimulating the children to avoid all of them.*

There are indications that low physical activity can contribute to the development of obesity among adolescents (Dencker et al., 2006), and that dietary restrictions combined with physical activity have a larger impact on weight loss than either one alone (Jakicic et al., 2005)*. Also, involvement of the family in the process, combined with the promotion of increments in energy expenditure, may contribute to the prevention of excessive weight gain.*

Logue et al., 2004 pointed out that groups which were encouraged to adopt healthier habits had lower rates of dropout compared to studies which focused mainly on weight loss, since the subjects in the former were not afraid of failure, which is common in weight loss programs. Therefore, during this intervention, we will not center our attention on obesity or its prevention. Individual activities will be developed based on making personal healthy choices, showed as positive, smart, and fashionable. The proposed intervention is based on key constituents of teenagers’ dietary habits, and it is expected to have a major impact on global energy balance.

1. **Goals and Objectives**

The main objective of this project is to evaluate the effect on BMI of a school-based program that discourages the consumption of all sweetened beverages, encourages the reduction in sugar intake, and encourages the increase in physical activity among adolescents and their families from a low socioeconomic area.

This proposal is being conducted as a joint venture with city hall. Those activities that prove effective during the research will be implemented by the municipality school district office.

This project relates with the following PAHEF line of work: *“Addressing nutrition and healthy lifestyles in the school curriculum. There is agreement that physical activity should be promoted in schools and in the general population and that the quality of foods and beverages offered in schools should be improved. However, how best to do this and in ways that can be scaled up?”*

Specific objectives are:

1. To compare total, lean, and fatty body mass variation in adolescents aged 11 to 14, from 7^th^ and 8^th^ grades from schools under intervention and from the ones not subjected to the intervention program.
2. To compare sweetened beverages consumption and sugar intake before and after the intervention in both groups of schools.
3. To evaluate the impact of the intervention on the selling of sweetened beverages in the cafeterias.
4. To compare change in family expenditures with sweetened beverages and sugar.
5. To ascertain the level of physical activity before and after the intervention.
6. **Project Design and Implementation Plan**

The project will be conducted with a sample of 7^th^ and 8^th^ grade students from public schools in the municipality of Duque de Caxias, located in the metropolitan area of Rio de Janeiro, Brazil, one of the poorest metropolitan areas in Brazil. The municipality of Duque de Caxias encompasses an area of 468 km^2^, with an estimated population of 850,000 inhabitants (IBGE, 2008). It is considered to be the seventh poorest municipality in the Rio de Janeiro state, with a proportion of 14.5% of its inhabitants living below the extreme poverty line (Rocha & Albuquerque, 2003). The district of Campos Elíseos, with 220,000 inhabitants and 35 municipal public schools was selected for this study based on the high level of poverty (Costa et al, 2008).

A cluster randomized controlled trial will be conducted in a sample draw from the 35 municipal public schools located in the district of Campos Elíseos (municipality of Duque de Caxias, state of Rio de Janeiro, Brazil). The period of intervention will be from March to November, during the school year.

In this area, most students in the public schools are from low socioeconomic level families. Children go to school either in the mornings (8:00am to noon) or in the afternoons (1pm to 5pm). Parents will be informed of the study and only those children with informed consent signed by their parents will be included. However, all of them are going to receive information and the promotional materials. The protocol will follow the Brazilian legislation regarding research involving humans.

**Sample size**

Sample size estimation is based on data from a previous study conducted in a city of the same metropolitan area for which the standard deviation in soda consumption was 1.49 cups per day. In order to be able to detect a difference of 0.5 glass of soda between the two groups with a power of 80% and a 5% significance level, the sample size needed is 140 children in each arm of the trial. This sample size also allows for the detection of a difference in BMI of about 1 unit.

Due to the cluster design (classrooms) and anticipating a 80% rate of agreement by the parents, 400 students are planned to be sampled. Schools, instead of classrooms, will be randomized to reduce contamination during the intervention. Schools will be ranked based on prevalence of overweight plus obesity, and randomization will be generated balancing the groups by body mass index at baseline. Children will be sampled according to the flowchart 1.

**Schools with 7^th^ and 8^th^ grades**

**(n=35)**

**Sample of one classroom per school**

**(n=20)**

(n=20 classrooms of about 20 children each)

**Schools randomized (n=20)**

**Intervention (n= 10 classrooms)**

200 children and parents

**Control (n= 10 classrooms)**

200 children and parents

Flowchart 1: Sampling design and randomization

**Intervention**

1. Discouraging sweetened beverages consumption:

The messages to be delivered have already been tested for understanding in two small groups of children of the same age and socioeconomic background of study participants. Beliefs and behaviors of children were already obtained from focus groups in order to orient activities and the production of printed materials to be given to participants (Sichieri et al. in press).

Education will be delivered via classroom activities**;** banners will be hanged promoting healthy drinking. Water bottles with the logo of the campaign will be given to children and schoolteachers.

A “pyramid of drinking” will be showed to communicate the message that water (the basis of the pyramid) should take prevalence over sugar-sweetened beverages (at the top of the pyramid). This graph was created and tested in the previous similar study mentioned above (Sichieri et al, in press). Moreover, there will be classroom quizzes and games using sugar and sweetened beverages as the theme.

Activities will be facilitated by trained research assistants. Furthermore, printed instructions and orientations on the facilitation process will support the assistants’ efforts. The activities will require 20 to 30 minutes, and teachers will be encouraged to reiterate the message during their lesson. The goal is to promote ten one-hour sessions of activity for each class.

Also, a musician, experienced in this kind of activity will help each class to collectively compose simple songs related to reducing the consumption of sugar and sugar-sweetened carbonated beverages. This musical activity has already been tested and it is welcomed by both children and teachers; it will be conducted during three one-hour sections in each classroom.

b) Encouraging the reduction of sugar intake:

To encourage the reduction of sugar addition in coffee, for example, in order to make coffee less bitter, individuals will be advised to add milk to it. To promote the reduction of sugar addition in other beverages, like fruit juices, children will be advised to add water, and stimulated to savor the fruits’ flavor. Decrease in the intake of sweetened juices will be encouraged by motivating the reduction of the portions and the dilution with water. Sample tasting and simulation of organoleptic evaluations of these alternative preparations will take place in the intervention classes.

c) Increasing physical activity

Children will be stimulated to increase everyday activities such as walking and playing games at home and school. They will be encouraged to engage in as many physical activities as possible. Balls, ropes, and “petecas” (type of Brazilian shuttlecock) will be available to be lent out to the children.

They will be asked to register the amount of time spending watching TV/computer/games (our previous data indicate a mean of 4 hours per day) during one weekday and one weekend day per week.

They will be encouraged to exchange TV viewing for games, and children reporting about one hour reduction in TV or computer use will receive simple gifts related to outdoor activities. These numbers will be added for each class, and a two-month competition will take place to stimulate the achievement of greater reductions. Values for all ten intervention classes will be shared with the students and used in math classes.

Parents will also be stimulated to increase the practice of physical activities. We already developed and tested a booklet to help adults to do home based exercises using balls and ropes. Parents will receive a booklet with instructions and figures describing exercises to be done at home, in sessions of 30 minutes including a 5 minute warm up, five days per week. Exercise intensity is classified as moderate (Ainsworth et al., 2000). Also, activities with parents and family members such as walking around the neighborhood on weekends will be promoted. Since our previous experience about meeting with parents was not successful, we will be sending home fliers and a fridge magnet to encourage cutting down on sodas and increasing the practice of physical exercises.

Track and field competitions among children and classes will be promoted.

d) Increasing knowledge regarding the project topics (soda intake, sugar intake, and physical activity):

To encourage the inclusion of these topics in the classes’ activities; for example:

Science: Dilution and saturation of liquids (sugar proportion); the role of sugar in metabolism; the importance of weight regulation to health; the principles of a healthy diet; the importance of physical activity to health and development; development of a “Science Fair” on specific topics.

Mathematics: Fractions, volume measurements, money, graphs (statistics).

Language: Elaboration of a diary regarding food intake and physical activity; compositions about the topics (for example in the format ‘pros and cons’); poetry contests and compositions about the subject.

Art education: Elaboration of projects with recycled material; activities involving music and drawing contests.

The control group will receive two one-hour general sections on health issues and general advice on healthy diets.

1. **Monitoring and Evaluation**

Independent variables:

a) Beverage intake measurement:

Children soda, sweetened beverage, and milk intake will be measured through one-24 hour recall and one specific frequency questionnaire at baseline and at the end of the trial. Drinking intake based on 24-hour recall for each class before and after intervention will be compared. Usual frequency of intake of beverages will use a short questionnaire with the previous month as the time frame. The questionnaire includes sugar-sweetened carbonated beverages (regular and diet), other sugar-sweetened beverages (regular and diet), milk, 100% fruit juices (referred from now on simply as fruit juice), canned and packed fruit juices, and powdered flavored beverages containing sugar.

The 24-hour recall and answers to the short questionnaire will be obtained by nutritionists during in-person interviews with the children at school.

b) Sugar intake:

Parents will answer how much sugar is bought monthly in the household at beginning and at the end of the study. Children will be asked how often they add sugar to specific beverages at beginning and at the end of the study. The estimated amount of sugar added to beverages will be obtained in the 24-hour recall.

c) Physical activity

A questionnaire about the practice of usual physical activities will be applied to the students at baseline and at the end of the study.

d) Knowledge on health effects of eating habits

Students will answer questions about diet and health at beginning and at the end of the study.

Main outcomes:

a) Mean body mass index (BMI=weight/height^2^) change. Anthropometric measurements will be taken at the beginning and at the end of the study. Height (without shoes) will be measured to the nearest 0.1 cm using portable stadiometer. Weight (in light clothing) will be measured to the nearest 0.1 kg on portable digital scales.

b) Prevalence of overweight and underweight: overweight and underweight will be defined using the BMI cutoffs proposed by Cole et al. (2000) and Cole et al (2007).

c) Mean lean and fatty body mass proportions: measured by portable electrical bioimpedance bean (Tanita body composition monitor BC-552).

**Statistical analysis**

Baseline characteristics of the two groups are going to be compared using Student’s t-test or the Chi-square test. Data on those who completed the study is going to be compared (baseline vs. after intervention) using paired t-test. Intention to treat analysis will be also performed using longitudinal analysis taking into account the cluster (classes) effect through mixed models (Singer & Wilett, 2003).

**References**

Alvarez MM, Vieira ACR, Moura AS, Veiga GV. Insulin resistance in Brazilian adolescent girls: association with overweight and metabolic disorders. *Diabetes Res Clin Pract* 2006; 74 (2): 183-88.

[Andrade RG](http://www.ncbi.nlm.nih.gov/entrez/query.fcgi?db=pubmed&cmd=Search&term="Andrade+RG"%5BAuthor%5D), [Pereira RA](http://www.ncbi.nlm.nih.gov/entrez/query.fcgi?db=pubmed&cmd=Search&term="Pereira+RA"%5BAuthor%5D), [Sichieri R](http://www.ncbi.nlm.nih.gov/entrez/query.fcgi?db=pubmed&cmd=Search&term="Sichieri+R"%5BAuthor%5D). Food intake in overweight and normal-weight adolescents in the city of Rio de Janeiro. Cad Saude Publica 2003; 19: 1485-95.

Ainsworth BE, Haskell WL, Whitt MC, Irwin ML, Swartz AM, Strath SJ, et al. Compendium of physical activities: an update of activity codes and MET intensities. *Med Sci Sports Exerc* 2000; 32(9 Suppl): S498-504.

Atkinson RL, Nitzke SA. School based programmes on obesity. *BMJ* 2001; 323 (7320): 1018-19.

Basset MT, Perl S. Obesity: the public health challenge of out time. *Am J Public Health* 2004; 94: 1477.

Berkey CS, Rockett HR, Field AE, Gillman MW, Colditz GA. Sugar-added beverages and adolescent weight change. *Obes Res* 2004; 12**(5)**: 778-88.

Cavadini C, Siega-Riz AM, Popkin BM. US adolescent food intake trends from 1965 to 1996. *Arch Dis Child* 2000; 83(1):18-24.

Cole TJ, Bellizzi MC, Flegal KM, Dietz WD. Establishing a standard definition for child overweight and obesity worldwide: International Survey. *BMJ* 2000; 320(7244): 1240-3.

[Cole TJ](http://www.ncbi.nlm.nih.gov/sites/entrez?Db=pubmed&Cmd=Search&Term="Cole TJ"%5BAuthor%5D&itool=EntrezSystem2.PEntrez.Pubmed.Pubmed_ResultsPanel.Pubmed_DiscoveryPanel.Pubmed_RVAbstractPlus), [Flegal KM](http://www.ncbi.nlm.nih.gov/sites/entrez?Db=pubmed&Cmd=Search&Term="Flegal KM"%5BAuthor%5D&itool=EntrezSystem2.PEntrez.Pubmed.Pubmed_ResultsPanel.Pubmed_DiscoveryPanel.Pubmed_RVAbstractPlus), [Nicholls D](http://www.ncbi.nlm.nih.gov/sites/entrez?Db=pubmed&Cmd=Search&Term="Nicholls D"%5BAuthor%5D&itool=EntrezSystem2.PEntrez.Pubmed.Pubmed_ResultsPanel.Pubmed_DiscoveryPanel.Pubmed_RVAbstractPlus), [Jackson AA](http://www.ncbi.nlm.nih.gov/sites/entrez?Db=pubmed&Cmd=Search&Term="Jackson AA"%5BAuthor%5D&itool=EntrezSystem2.PEntrez.Pubmed.Pubmed_ResultsPanel.Pubmed_DiscoveryPanel.Pubmed_RVAbstractPlus). Body mass index cut offs to define thinness in children and adolescents: international survey.*BMJ* 2007; 335(7612): 194-7

Costa, RS, Pereira, R A,Vasconcellos, MTL,Veiga, GV da ; Marins, VMR de, Jardim, B , Gomes, FS, Sichieri, R. Associação entre fatores socioeconômicos e insegurança alimentar: estudo de base populacional na Região Metropolitana do Rio de Janeiro, Brasil. Revista de Nutrição, 2008 in press.

Dencker M, Thorsson O, Karlsson MK et al. Daily physical activity related to body fat in children aged 8–11 years. *J Pediatr* 2006; 149(1): 38–42.

Gortmaker SL, Peterson K, Wiecha J, et al. Reducing obesity via a school-based interdisciplinary intervention among youth. *Arch Pediatr Adolesc* 1999; 153(4): 409-18.

Grimm GC, Harnack L, Story M. Factors associated with soft drink consumption in school-aged children*. J Am Diet Assoc* 2004; 104(8): 1244-9.

[Gibson S](http://www.ncbi.nlm.nih.gov/sites/entrez?Db=pubmed&Cmd=Search&Term="Gibson S"%5BAuthor%5D&itool=EntrezSystem2.PEntrez.Pubmed.Pubmed_ResultsPanel.Pubmed_RVAbstractPlus), [Neate D](http://www.ncbi.nlm.nih.gov/sites/entrez?Db=pubmed&Cmd=Search&Term="Neate D"%5BAuthor%5D&itool=EntrezSystem2.PEntrez.Pubmed.Pubmed_ResultsPanel.Pubmed_RVAbstractPlus). Sugar intake, soft drink consumption and body weight among British children: Further analysis of National Diet and Nutrition Survey data with adjustment for under-reporting and physical activity. *Int J Food Sci Nutr* 2007;58(6):445-60.

Instituto Brasileiro de Geografia e Estatística. Pesquisa de Orçamentos Familiares 2002 – 2003: antropometria e análise do estado nutricional de crianças e adolescentes no Brasil [Online]. 2006 [cited 2008 May 27]. Available from: URL: http://www.ibge.gov.br

Jakicic JM, Otto AD. Physical activity considerations for the treatment and prevention of obesity. *Am J Clin Nutr* 2005; 82(1Suppl): 226S–9S.

James J, Thomas P, Cavan D, Kerr D. Preventing childhood obesity by reducing consumption of carbonated drinks: cluster randomised controlled trial. *BMJ* 2004; 328(7450): 1237.

Johnson CC, Li D, Galati T, Pedersen S, Smyth M, Parcel GS. Maintenance of the classroom health education curricula: results from the CATCH-ON study. *Health Educ Behav* 2003; 30 (4): 476-88.

Katz DL, O’Connell M, Njike VY, Yeh MC, Nawaz H. Strategies for the prevention and control of obesity in school setting:systematic review and meta-analysis. *Int J Obes* 2007:1-11.

[Levy-Costa RB](http://www.ncbi.nlm.nih.gov/entrez/query.fcgi?db=pubmed&cmd=Search&term="Levy-Costa+RB"%5BAuthor%5D), [Sichieri R](http://www.ncbi.nlm.nih.gov/entrez/query.fcgi?db=pubmed&cmd=Search&term="Sichieri+R"%5BAuthor%5D), [Pontes NS](http://www.ncbi.nlm.nih.gov/entrez/query.fcgi?db=pubmed&cmd=Search&term="Pontes+Ndos+S"%5BAuthor%5D), [Monteiro CA](http://www.ncbi.nlm.nih.gov/entrez/query.fcgi?db=pubmed&cmd=Search&term="Monteiro+CA"%5BAuthor%5D). Household food availability in Brazil: distribution and trends (1974-2003). *Rev Saúde Pública* 2005; 39 (4): 530-40.

[Logue EE, Jarjoura DG, Sutton KS, Smucker WD, Baughman KR, Capers CF*.*](http://www.ncbi.nlm.nih.gov/entrez/query.fcgi?cmd=Retrieve&db=pubmed&dopt=Abstract&list_uids=15483215&query_hl=20)Longitudinal relationship between elapsed time in the action stages of change and weight loss. *Obes Res* 2004; 12(9):1499-508.

Ludwig DS, Peterson KE, Gortmaker SL. Relation between consumption of sugar-sweetened drinks and childhood obesity: a prospective, observational analysis. *Lancet* 2001; 357 (9255): 505-8.

McGarvey E, Keller A, Forrester M, Williams E, Seward D, Suttle DE. Feasibility and benefits of a parent-focused preschool child obesity intervention. *Am J Public Health* 2004; 94(9): 1490-5.

Malik VS, Schulze MB, Hu FB. Intake of sugar-sweetened beverages and weight gain: a systematic review. [*Am J Clin Nutr*](javascript:AL_get(this, 'jour', 'Am J Clin Nutr.');) 2006;84(2):274-88.

[Monteiro CA](http://www.ncbi.nlm.nih.gov/entrez/query.fcgi?db=pubmed&cmd=Search&term="Monteiro+CA"%5BAuthor%5D), [Conde WL](http://www.ncbi.nlm.nih.gov/entrez/query.fcgi?db=pubmed&cmd=Search&term="Conde+WL"%5BAuthor%5D), [Popkin BM](http://www.ncbi.nlm.nih.gov/entrez/query.fcgi?db=pubmed&cmd=Search&term="Popkin+BM"%5BAuthor%5D). The burden of disease from undernutrition and overnutrition in countries undergoing rapid nutrition transition: a view from Brazil. *Am J Public Health* 2004; 94(3): 433-4.

Newby PK, Peterson KE, Berkey CS, Leppert J, Willett WC, Colditz GA. Beverage consumption is not associated with changes in weight and body mass index among low-income preschool children in North Dakota. *J Am Diet Assoc* 2004; 104**(7)**: 1086-94.

Oliveira CL, Veiga GV, Sichieri R. Anthropometric markers for cardiovascular disease among overweight adolescents. *Nutr Res* 2001; 21: 1335-45

Rocha S, Albuquerque RC. Geografia da pobreza extrema e vulnerabilidade à fome. In: Instituto Nacional de Altos Estudos. Seminário fome e pobreza. Rio de Janeiro: INAE; 2003.

Sahota P, Rudolf MC, Dixey R, Hill AJ, Barth JH, Cade J. Evaluation of implementation and effect of primary school based intervention to reduce risk factors for obesity. *BMJ* 2001; 323: 1027-9.

Sichieri R. Epidemiologia da obesidade. Rio de Janeiro: EdUERJ; 1998..

Sichieri R, Trotte AP, Souza RA,,Veiga GV- School randomized trial on prevention of excessive weight gain by discouraging students from drinking sugar-sweetened carbonated beverages. Public Health Nutrition- in press.

Singer JD, Willett JB, editors. Applied longitudinal data analysis – modeling change and event occurrence. New York: Oxford University Press; 2003.

Stice H, Shaw H, Marti N. A Meta-Analytic Review of Obesity Prevention Programs for Children and Adolescents: The Skinny on Interventions that Work. *Psychol Bull* 2006; 132 (5): 667–91.

[Striegel-Moore RH, Thompson D, Affenito SG, Franko DL, Obarzanek E, Barton BA, .](http://www.ncbi.nlm.nih.gov/entrez/query.fcgi?db=pubmed&cmd=Retrieve&dopt=AbstractPlus&list_uids=16492426&query_hl=3&itool=pubmed_docsum) Correlates of beverage intake in adolescent girls: the National Heart, Lung, and Blood Institute Growth and Health Study. *J Pediatr*2006; 148 (2): 183-7.

Sutton A. Letter Childhood obesity and consumption of fizzy drinks: Play outside to reduce childhood obesity. *BMJ* 2004; 329(7456): 54.

Stice E, Shaw H, Marti CN. A Meta-Analytic Review of Obesity Prevention Programs for Children and Adolescents: The Skinny on Interventions that Work. *Psychol Bull* 2006; 132(5): 667-91.

Thomas H. Obesity prevention programs for children and youth: why are their results so modest? *Health Educ Res* 2006; 21 (6): 783–95.

University of York: NHS Centre for Reviews and Dissemination. Effective health care: the prevention and treatment of childhood obesity. London: *Royal Society of Medicine*, 2002; 7 (6).

Van Wymelbeke V, Beridot-Therond ME, de La Gueronniere V, Fantino M. Influence of repeated consumption of beverages containing sucrose or intense sweeteners on food intake. *Eur J Clin Nutr* 2004; 58(1): 154-61.

Veiga GV, da Cunha AS, Sichieri R. Trends in overweight among adolescents living in the poorest and richest regions of Brazil. *Am J Public Health* 2004; 94 (9): 1544-8.

[Warren JM, Henry CJ, Lightowler HJ, Bradshaw SM, Perwaiz S.](http://www.ncbi.nlm.nih.gov/entrez/query.fcgi?cmd=Retrieve&db=pubmed&dopt=Abstract&list_uids=14695360&query_hl=17) Evaluation of a pilot school programme aimed at the prevention of obesity in children. *Health Promot Int* 2003; 18(4): 287-96.

World Health Organization. Expert Committee. Physical Status: The use and Interpretation of Anthopometry [Thecnical Report Series 854]. Geneva; 1995.

**Organizational Capacity (1-2 pages)**

The Social Medicine Institute of the State University of Rio de Janeiro has 32 years of experience in research in the area of Public Health and social determinants of health in Brazil. The CEPESC – The Center of Research in Collective Health, the administrative arm of the Social Medicine Institute, currently oversees 88 projects in the field of Public Health. These projects have as principal investigators the 35 research employees of the Institute, as well as researchers from other institutions.

The mission of the Institute is to conduct research and to act as a consultant to the Brazilian Ministry of Health in fields such as: administration of the National Health System, urban and domestic violence, public polices in administration and health, in gender and health, and also in the field of Nutrition.

The Institute has 3 departments: Epidemiology, Administration, and Social Sciences, and it teaches 3 post graduate courses: Collective health, Administration of health, and Epidemiology for the health services. Research in Nutrition has been focused on obesity, food intake by adolescents and methodology of food intake estimation.

The principal investigator in this proposal recently finished a community trial on weight change and reduction in the consumption of carbonated drinks and the lessons learn in the trial are being implemented in this project. We choose not concentrate on school curriculum because many studies, as indicated in the introduction, achieved good results in changing knowledge but not in curbing the epidemic of obesity. We already accumulated experience about do nots, such as: do not target obesity, do not expect to bring the families to the school, do not aim to cut dow on sodas, but to exchange with low sugar products. Therefore this proposal has better chance to prove that obesity can be reduced among adolescents.
