# Supplementary figures and images for: Effectiveness of a Randomized School-Based Intervention Involving Families and Teachers to Prevent Excessive Weight Gain among Adolescents in Brazil
Source: PLoS One. 2013 Feb 25;8(2):e57498. doi: 10.1371/journal.pone.0057498 (PMC3581462; doi:10.1371/journal.pone.0057498)

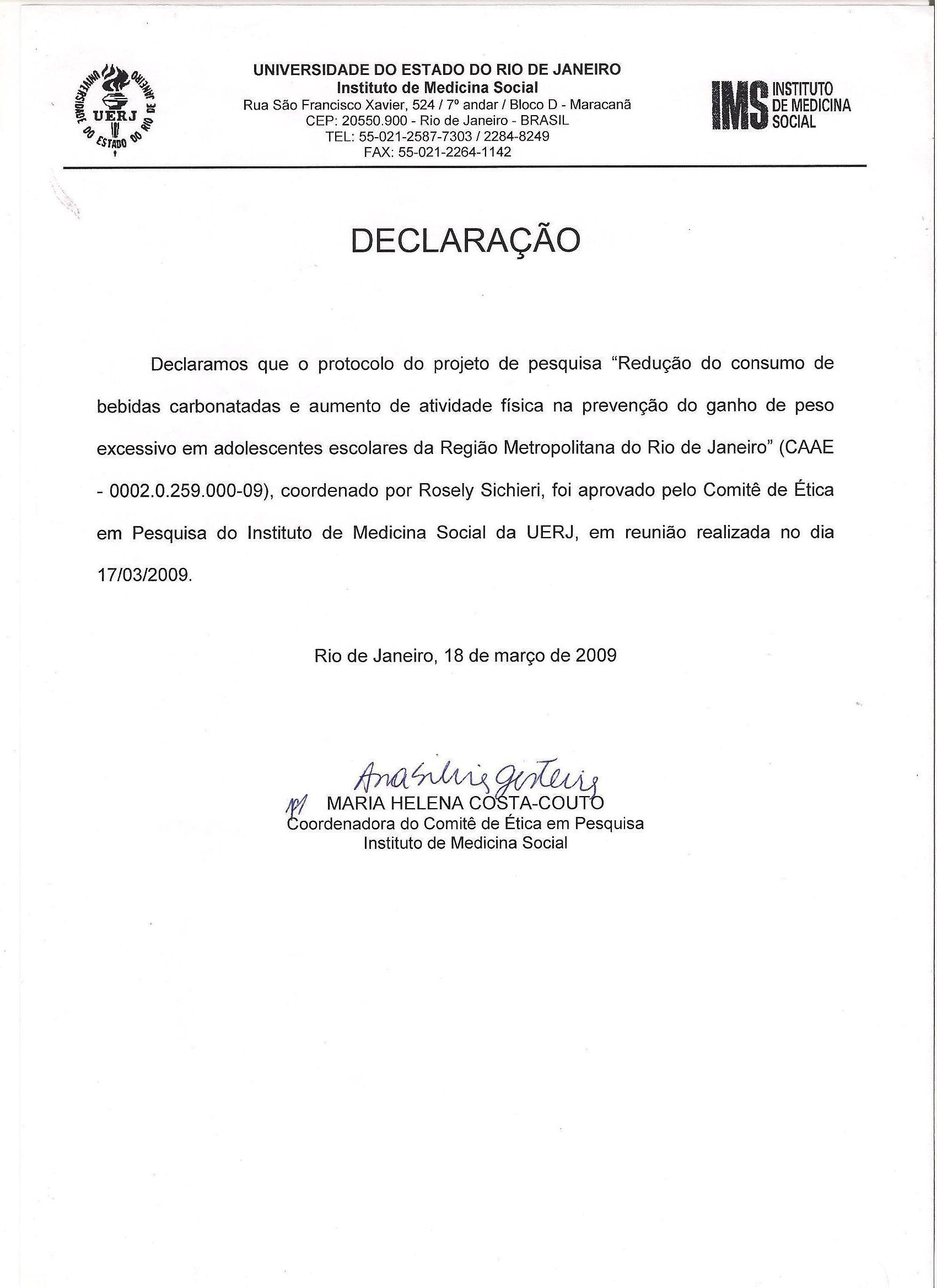

Supplement: Doc S1 — Approval of the Ethics Committee. (JPG) [file pone.0057498.s003.jpg]
