# Supplementary material for: Effectiveness of a Randomized School-Based Intervention Involving Families and Teachers to Prevent Excessive Weight Gain among Adolescents in Brazil
Source: PLoS One. 2013 Feb 25;8(2):e57498. doi: 10.1371/journal.pone.0057498 (PMC3581462; doi:10.1371/journal.pone.0057498)
Supplement: Doc S2 — Description of the intervention sessions. (DOC) [file pone.0057498.s004.doc]

The intervention had focus on incentive changes in food consumption. To this end, at the school year of 2010, there were conducted nine sessions of approximately one hour in the classroom, conducted by trained nutritionists, which include games, theater and play. These activities are described in detail below.

The activities were aimed to discouraging the consumption of sugar-sweetened beverages, reducing the consumption of sugar; replace snacks based on processed food (especially cookies) for food and fresh fruits. In order to reinforce the messages and activities developed with children, there were extended to the family plan, by sending folders, recipes and gifts at each activity held. Teachers were also included in all activities and encouraged to continue the intervention performed in each classroom activities, aimed to expanding students' knowledge on the topics of the project. For each session were developed: 1) activities related to the subject, conducted by nutritionists at the school during regular class time, 2) sending folders explaining the intervention program and suggesting the involvement of the family of students, 3) strategies for teachers include school activities related to healthy eating habits, such as lesson plans specifically designed for this purpose.

The first session was performed in March 2010 and the subject was "Healthy Eating". The theme of the first intervention was comprehensive in order to establish the first contact between students and members of the project. At this time, students and teachers were presented to the project and the activities that would be developed throughout the school year in classes.

In this session, a video with duration of 6 minutes was played. Briefly, the video, at cartoon format, depicts two individuals who go to the supermarket and have to decide to listen the counsel from an angel, that encourages healthy food choices, or a devil, which encourages the purchase of unhealthy food, mostly industrialized.

Then, there was discussion about the video, and after this, a list of words related to the film was given to the students, and they must to look in the dictionary the meaning of each one. As a group, they create a sentence about the topic discussed. To do this, they received a gift of pencils with the logo of PAPPAS - Eat Food.

Parents and guardians were also presented to the project through a folder which describes the actions of PAPPAS in school at 2010 school year and requested the support of them to achieve the objectives.

At April 2010, it was performed the second session with the classes, using the theme “Indian Day”, since the activity happened just days after the commemoration of this date.
Children watched a video with pictures, simultaneously with the audio on "The Legend of Cassava." In short, this is a Brazilian legend that explains the origin of this tubercle by the Indians.

Was subsequently held discussion on the topic, and then, students were divided into groups and reprinted the story according to figures given. Finally, students and teachers tasted a cassava and coconut cake, prepared by PAPPAS team, with a little added sugar. It was then launched a challenge for students: stay a week without drinking sweetened beverages. The cassava cake recipe was sent to parents / guardians through a folder. In addition, a greeting card was sent by Mother's Day. That card had a challenge to parents: do not buy sweetened beverages for a month in order to corroborate the challenge to students.

Teachers received a support material in order to continue the topic addressed in the intervention, with the class. This material was prepared with the aid of an elementary school teacher, and included in the proposed objectives, justification, prior knowledge required and suggested activities on the topic addressed by PAPPAS team. At all of the following interventions, this material was delivered to teachers.

The third session was realized at May 2010 and had the theme: “Sugar at food”. It was made a demonstration of the amount of sugar present in sweetened beverages, using three cups of sugar with different amounts (relative to a 2-liter bottle, a can, a bottle of 600 mL and a glass of sweetened beverages). A package containing sugar was used to demonstrate the amount of sugar present in 5 bottles of soda.

As the children were challenged to stay a week without drink sweetened beverages (at the previous session), was made a quick calculation showing the amount of sugar that has been avoided and also the quantity of PET bottles is no longer consumed, if the student has made the challenge.
Finally, class was divided into two groups and was held a quiz with question and answer about sugar.

At June 2010, the fourth session was conducted, about “The marriage of Rice and Bean”. Initially, it was made a conversation about the amino acids at the combination of rice and beans, and was presented bags containing different types of beans and legumes to the students. Also, there was a puppet theater of the marriage of rice and beans. In return for parents, was delivered a refrigerator magnet about the topic and a brochure.

The fifth session was performed at July 2010 about “Fruits”. It was highlighted the importance of replacing the consumption of cookie for fruits. Three activities were undertaken:
1) Students should, blindfolded, guessing the fruit that was offered to them, through touch, smell, taste and feel of food in the mouth; 2) It was given masks of fruit to the students and they participated to a parade, accompanied by narration of the properties of each fruit; 3) They tasted some chopped fruit. In return for parents, was sent a folder.

At August 2010, the session performed was about the water, called “Super Water”. Initially, it was promoted a conversation about the importance of water consumption for the human body as well as their rationing, to preserve it. At the end of the exhibition, became a professional of physical education staff dressing as a superhero, the Super Water, with the sound of the theme song for the movie Superman. The Super Water gave a glass with the name of each student. Finally, children create superpowers for the Super Water, based on the benefits of water, previously mentioned.

The seventh activity was developed on September 2010, about “Cookies”. It was recalled the importance of reducing consumption of cookies and sweetened beverages and increased consumption of fruits. It stimulated the consumption of school meals, which offers a daily fruit as dessert.

It was made a board game and a word search on all issues encompassing themes. Finally, the class was delivered to a banner with the best powers created for the Super Water and a photo of the class with the super hero. In return for the parents, was handed a folder with super powers chosen by the class for the Super Water.

The penultimate activity was performed at October 2010, called “Mini-market”.

The class was divided into groups and each received a fictitious notes equivalent to $ 20 and should buy what they wanted with the stipulated amount. Among the options was fruits and

vegetables, cookies, snacks, water, industrialized juices, soft drinks and other kinds of sweetened beverages, and food dishes. All food purchased by each group were recorded on the blackboard, and the points were computed for each one. Natural products received positive points and negative points were given to industrialized products. Then the students were analyzed with the labels on packaging, highlighting the use of dyes and flavorings. We proposed a new challenge for students: do not eat sweetened beverages and cookies for a month.

Finally, the last activity performed was about “Advertisements”.

Conversation was carried on the existing food advertisements, noting that in most cases, these advertisements are about industrialized foods and aimed at children. Some of these advertisements were shown and then the class was divided into groups. Each group was responsible for preparing an advertising (television or magazine /newspaper), using the tricks used by the media. As this was the last action performed, there was a small graduation, where acts of participation were distributed.

Students received a keychain with the logo of PAPPAS, as thanks for participating.
In return for the parents, was handed a folder with an appreciation for the participation and revenue, as well as a refrigerator magnet with logo PAPPAS. For teachers, was given the same key ring and a folder of thanks.
